# Effect of Microwave Pretreatment of Seeds on the Quality and Antioxidant Capacity of Pomegranate Seed Oil

**DOI:** 10.3390/foods9091287

**Published:** 2020-09-14

**Authors:** Tafadzwa Kaseke, Umezuruike Linus Opara, Olaniyi Amos Fawole

**Affiliations:** 1 Postharvest Technology Research Laboratory, Department of Food Science, Faculty of AgriSciences, Stellenbosch University, Stellenbosch 7602, South Africa; tafakaseqe@gmail.com; 2 Postharvest Technology Research Laboratory, Department of Horticultural Sciences, Faculty of AgriSciences, Stellenbosch University, Stellenbosch 7602, South Africa; 3 Postharvest Research Laboratory, Department of Botany and Plant Biotechnology, University of Johannesburg, Johannesburg 2006, South Africa

**Keywords:** pomegranate seeds, oil, microwave pretreatment, total phenolic content, antioxidant capacity

## Abstract

Microwave pretreatment of oilseeds is a novel technique used to enhance oil nutraceutical properties. In this study, the effect of microwave pretreatment of seeds was investigated on pomegranate seed oil quality attributes including oil yield, yellowness index, refractive index, peroxide value, *ρ*-anisidine value, total oxidation value, conjugated dienes, total phenolic content, total carotenoids content, phytosterol composition, fatty acid composition, 2,2-diphenyl-1-picrylhydrazyl (DPPH) radical scavenging capacity, and ferric reducing antioxidant power (FRAP). The seeds of three different pomegranate cultivars (‘Acco’, ‘Herskawitz’, and ‘Wonderful’) were microwave heated at 261 W for 102 s. Pomegranate seeds microwave pretreatment enhanced oil yield, yellowness index, total carotenoids content, total phenolic content, FRAP and DPPH radical scavenging capacity, despite an increase in conjugated dienes, and peroxide value. Palmitic acid, oleic acid, linoleic acid, saturated, and monosaturated fatty acids were increased after pomegranate seeds microwave pretreatment, whilst the levels of punicic acid and β-sitosterol were reduced. Nevertheless, the refractive index, the ratio of unsaturated to saturated fatty acid of the extracted oil were not significantly (*p* > 0.05) affected by pomegranate seeds microwave pretreatment. Principal component analysis and agglomerative hierarchical clustering established that ‘Acco’ and ‘Wonderful’ oil extracts from microwave pretreated PS exhibited better oil yield, whilst ‘Herskawitz’ oil extracts showed higher total carotenoids content, total phenolic content, and antioxidant capacity.

## 1. Introduction

The demand for the use of natural products in preventing chronic and degenerative diseases has increased in recent decades, driven by increased consumer health awareness [1]. Attention has been given to functional foods that provide both nutritional functions and health benefits. Pomegranate fruit is rich in both nutritional and biological properties [2]. The fruit has been cultivated since ancient times throughout the Mediterranean region, mainly for its nutritional and pharmacological value [3]. Pomegranates have been used in the treatment of sore throats, coughs, ulcers, urinary infections, intestinal worms, digestive disorders, skin disorders, and arthritis for centuries [4]. Apart from being consumed as fresh fruits, pomegranates may be processed into various products such as juice, jam, wine, syrup, among other products. The fruit contains seeds that range between 37 and 143 g/kg of the total fruit weight depending on factors such as cultivar, growing region, growing conditions, and maturity stages [5,6]. Pomegranate seeds (PS) have oil that range between 12% to 20% (dry weight basis) and is a good source of bioactive compounds such as punicic acid, tocopherols, phenols, sterols, and carotenoids [3,7].

Epidemiological studies have revealed that pomegranate seed oil (PSO) has biological properties related to the prevention of microbial growth, lipoperoxidation, skin photoaging, cancer, diabetes, and obesity that are linked to the bioactive compounds [7,8]. In line with the biological activities, the oil can be used as a functional ingredient in nutraceutical, pharmaceutical, and functional foods preparations [9]. Despite the wealth of health benefits in PSO, the seeds are still regarded as waste after processing the fruits into juice and other products, thereby increasing the total pomegranate fruit postharvest losses. In some instances, the seeds are used as stock feed [10]. From an industrial and health perspective, valorization of the PS into oil presents a more valuable alternative utilization of the postharvest waste.

Among other conventional seed oil extraction techniques, such as cold pressing and supercritical carbon dioxide, extraction using organic solvents is the best technique with regards to oil extraction efficiency [11]. Needless to say, the use of organic solvents such as hexane has become unpopular due to its hazardous effects on humans and the environment [11,12]. Consequently, the avoidance or reduction in the use of hexane as an extraction solvent has become a requirement for the food industry [13]. Alternatively, short-chain alcohols such as ethanol are promising solvents in seed oil extraction. Ethanol is a less hazardous, bio-renewable, and cheaper organic solvent [14]. The high polarity of ethanol provides it with the ability to extract polar bioactive compounds, such as the phenolic compounds, and therefore oil extracted with ethanol has better biological activities [15]. The main drawback in using ethanol is that it produces low oil yield [16,17]. Therefore, treatment of the oilseeds before oil extraction is important for improving the oil extraction efficacy of ethanol.

The treatment of seeds with microwaves before oil extraction has received great interests due to obvious advantages including uniform energy delivery, high thermal conductivity to the interior of the material, energy saving, and precise process control [18]. The application of microwave radiation to seeds result in direct interaction of the electromagnetic waves with the polar oxygen group from the seeds moisture [19]. This results in rapid heating and evaporation of the moisture in the seeds, thereby creating an internal pressure that causes the rapture of seed matrices [20]. The seeds microstructure alterations facilitated by microwave heating increases the interaction of the extraction solvent with the intracellular materials and enhances the lipids and bioactive compounds mass transfer into the extraction solvent [21]. Zhang and Jin [22], Li et al. [23], Porto et al. [24], and Güneşer and Yilmaz [25] have reported an improvement in oil yield and bioactive compounds recovery after microwave pretreatment of the camellia oleifera, yellow horn, moringa, and orange seed, respectively.

In order to establish whether microwave pretreatment adds value or not to pomegranate seed oil, it is important to investigate different cultivars. Cultivar significantly influences the quality of seed oil from the perspective of genetic characteristics variation [26]. Therefore, the effect of microwave pretreatment on the quality of the extracted oil may also vary with cultivar. To optimize the economic benefits, cultivars with better oil quality after seeds microwave pretreatment are valuable to food processors. However, information about the application of microwave irradiation on seed from different cultivars to establish variation in the quality of the extracted oil is limited.

Therefore, this study aimed to investigate the effect of microwave pretreatment of seeds on the quality and antioxidant capacity of PS oil extracted from three pomegranate cultivars.

## 2. Materials and Methods

### 2.1. Experimental Material

Pomegranate fruits (cv. Wonderful, Herskawitz, Acco) free of quality defects were harvested at commercial maturity stage from a farm (33°48′0″ S, 19°53′0″ E) in Western Cape Province, South Africa between February and April during the 2019 season. Pomegranate seeds (PS) extracted from the fruits were thoroughly cleaned before drying in an oven at 55 ± 2 °C for 24 h [27]. The dried seeds were stored at 4 ± 2 °C before use [28].

### 2.2. Sample Moisturizing

The moisture content of PS was measured in a moisture analyzer at 100 °C (DBS60-3, KERN, Balingen, Germany). The procedure of PS moisturizing was done following the method described by Rekas et al. [29]. PS (200 g) was sprayed with the pre-calculated amount of water, thoroughly mixed, sealed in zipped polyethylene bags, and equilibrated at 4 ± 2 °C for 48 h. This procedure was applied to moisturize the seeds to obtain a moisture content of 6% before microwave pretreatment. Water is a polar molecule and an important heat transfer medium during seeds microwave pretreatment and therefore PS moisturizing was vital [30]. A mass balance was used to calculate the amount of water to be added to the PS to obtain 6% moisture content. After 48 h of equilibration, the PS moisture content was checked in order to verify moisture homogeneity in the samples.

### 2.3. Microwave Pretreatment

#### 2.3.1. Equipment Calibration

A 2450 MHz domestic microwave oven (Model: DMO 351, Defy Appliances, Cape Town, South Africa) with a nominal power of 900 W was used in the present study. The microwave power calibration was performed following the procedure described by Rekas et al. [29]. Briefly, 500 g of water was heated in a glass beaker (80 mm diameter) and the time of 10 ± 2 °C elevation of water temperature was measured. The procedure was done in triplicate. The microwave power absorbed by the water during the heating was calculated as:

(1) $$W=m_{w}C_{pw}\frac{\Delta T}{\Delta t}$$

where *W* is the power absorbed by the water (*W*), *m_w_* is the mass of water (kg), *C_pw_* is the specific heat (J/C kg), Δ*T* is the difference in temperature (°C), and Δt is the time (s). The absorbed power by the water was 261 W for the applied 40% microwave power.

#### 2.3.2. Pretreatment

Ground PS (30 g) of uniform particle size (<1 mm) were evenly spread in a glass petri dish (190 mm in diameter) inside the calibrated microwave. The seed powder was exposed to microwave irradiation at 2450 MHz and 261 W for 102 s. This condition was established in preliminary experiments using response surface methodology (RSM), which confirmed 261 W and 102 s as the optimum microwave conditions for higher oil yield and antioxidant activity (unpublished). The microwave treated PS powder was allowed to cool to ambient temperature and thoroughly mixed to ensure sample homogeneity. Each experiment was performed in triplicate.

### 2.4. Oil Extraction

An ultrasonic bath (Separation Scientific, Cape Town, South Africa) (700 W, 40 kHz and 25 L capacity) was used to extract the oil. The PS powder (20 g) was mixed with 100 mL ethanol in 500 mL plastic capped glass bottles. The samples were sonicated at 700 W, 40 ± 5 °C for 40 min before filtration through Whatman No. 1 filter paper and vacuum evaporation to recover the solvent (G3 Heidolph, Schwabach, Germany). Unmicrowaved PS powder was used as the control samples. Oil extractions were done twice on triplicated samples (*n* = 3). The yield of pomegranate seed oil (PSO) was calculated using Equation (2).

(2) $$\mathrm{PSO}\;\mathrm{yield}\;\left(\%\right)=\frac{M_{1}}{M_{2}}\times 100$$

where M_1_ and M_2_ are the mass of PSO and dry weight (dw) of the pomegranate seed powder, respectively. The extracted PSO samples were packed in brown bottles and stored at 4 ± 2 °C to minimize oxidation during analyses [31].

### 2.5. Pomegranate Seeds Microstructures Analysis

Scanning electron microscopy (SEM) studies assess changes in the PS morphology due to microwave treatment and were conducted using the field emission scanning electron microscope (FESEM) (Thermo Fisher Apreo, Hillsboro, OR, USA). The samples were mounted on aluminum stubs using a double-sided carbon tape before sputter-coating with a thin layer of gold (10 nm thick) using a gold sputter coater (EM ACE200, Leica, Wetzlar, Germany) to induce conductivity within the sample. A voltage of 2 kV was used to collect the images, which were recorded digitally.

### 2.6. Determination of PSO Quality Indices

#### 2.6.1. Refractive and Yellowness Index

A calibrated Abbe 5 refractometer (Bellingham + Stanley, Kent, United Kingdom) was used to measure refractive index (RI) at ambient condition (25 °C). PSO colour properties including L* (lightness) and b* (yellowness) measured using a calibrated Chromameter CR-410 (Konica Minolta, INC, Tokyo, Japan) were used to calculate yellowness index (YI).

(3) $$\mathrm{YI}=\frac{142.86b^{*}}{L^{*}}$$

#### 2.6.2. Peroxide Value, Conjugated Dienes, *ρ*-Anisidine Value and Total Oxidation Value

PSO peroxide value (PV) was determined using the modified ferrous oxidation-xylenol orange (FOX) method [32]. Conjugated dienes (K232) and trienes (K270) were analyzed according to the standard [33]. The *ρ*-anisidine value (AV) was measured in accordance with [34]. Total oxidation (TOTOX) value was calculated from the PV and AV using the equation [35].

(4) TOTOX=2PV+AV

### 2.7. Determination of Bioactive Compounds and Antioxidant Capacity

#### 2.7.1. Total Carotenoids Content and Total Phenolic Content

Total carotenoids content (TCC) was measured following the method described by Ranjith et al. [36]. Briefly, PSO (0.2 g) was dissolved in hexane (5 mL) and 0.5 mL of 0.5% (*w*/*v*) sodium chloride (NaCl) was added. The mixture was vortexed and centrifuged (Centrifuge 5810R, Eppendorf, Germany) at 4000 rpm for 5 min. The absorbance of the supernatant was measured at 460 nm using a UV spectrophotometer (Helios Omega, Thermo Scientific, Waltham, MA, USA). The results were reported as mg β-carotene/100 g of PSO. Total phenolic content (TPC) was determined using the Folin–Ciocalteu method [37]. The reaction mixture contained 200 µL of PSO methanol extracts, 250 µL of the Folin–Ciocalteau reagent and 750 µL of 2% (*w*/*v*) sodium carbonate, and 3 mL of distilled water. The reaction mixtures were incubated in the dark for 40 min after which their absorbances were measured at 760 nm using a UV spectrophotometer (Helios Omega, Thermo Scientific, Waltham, MA, USA), and the final results were expressed as milligram gallic acid equivalent per g PSO (mg GAE/g PSO).

#### 2.7.2. Phytosterol Composition

The phytosterol composition was determined following the method described by Fernandes et al. [6] with some modifications. PSO (100 mg) samples weighed in 15 mL glass vials were mixed with 2.5 mL of saponification reagent (94 mL of absolute ethanol, 6 mL of 33% (*w*/*v*) potassium hydroxide, 500 µL of 20% (*w*/*v*) ascorbic acid). A hundred microliters of 5α-Cholestane (1000 mg/L) in chloroform (internal standard) was added and the mixture vortexed before saponification in an oven at 60 °C for 1 h. After saponification, the samples were cooled in ice for 10 min, followed by the addition of 5 mL of distilled of water and 2 mL of chloroform. The mixture was vortexed before centrifugation at 3000 rpm for 4 min. The chloroform extracts (500 µL) were concentrated with a gentle stream of nitrogen to ± 200 µL. To 100 µL of the concentrated chloroform extracts, pyridine (100 µL), and N,O-Bis (trimethylsilyl) trifluoroacetamide (30 µL) were added, and the mixture was vortexed before derivatization at 100 °C for 1 h in an oven. The derivatized sterol fractions were analyzed using gas chromatography connected to mass spectrometry (GC-MS) (Thermo Scientific Co. Ltd., Milan, Italy). The samples were injected 100 °C and held for 2 min before they were heated to 250 °C at the speed of 7 °C/min. The temperature was maintained for 2 min. A split ratio of 5:1, and an injection volume of 1.0 μL were used. The flow rate of helium, the carrier gas was maintained at 1 mL/min. The detector was operated under electron impact mode at ionization energy of 70 eV, scanning between 40 and 650 m/z. For peak identification, a standard containing a mixture of sterols (β-sitosterol, stigmasterol and ergosterol) was used. Phytosterol compounds identification was done by comparing the retention times. The results were reported as mg/100 g of PSO.

#### 2.7.3. Radical Scavenging Ability

PSO antiradical activity was evaluated using 2,2-Diphenyl-1-picryl hydrazyl (DPPH) assay [38]. Briefly, PSO methanol extracts (100 µL) were added to 2.5 mL of 0.0004% (*w*/*v*) freshly prepared DPPH in 80% (*v*/*v*) methanol. The mixture was vortexed before incubation in the dark for 60 min. The absorbance of the remaining DPPH was measured using a UV spectrophotometer (Helios Omega, Thermo Scientific, Waltham, MA, USA) at 517 nm. The absorbance of DPPH in 80% methanol was measured as the negative control. The final result was expressed as mmol Trolox/g of PSO.

#### 2.7.4. Ferric Reducing Antioxidant Power

The ferric reducing antioxidant power (FRAP) of PSO methanol extracts was determined following the method described by Benzie and Strain [39]. Freshly prepared FRAP reagent consisting of 2.5 mL of 10 mM 2,4,6-Tri(2-pyridyl)-s-triazine (TPTZ) solution in 40 mM HCl, 2.5 mL of 20 mM FeCl_3_ and 25 mL of 0.3 M acetate buffer, pH 3.6 was warmed at 37 °C for 10 min. In total, 40 microliters of PSO methanol extracts were mixed with 200 µL distilled water and 1.8 mL FRAP reagent. The samples were incubated at 37 °C for 30 min before the absorbances were measured at 593 nm using a UV spectrophotometer (Helios Omega, Thermo Scientific, Waltham, MA, USA). Trolox was used to prepare the standard curve (5-100 mM), and the final results were expressed as mmol Trolox/g of PSO.

### 2.8. Fatty Acid Composition

Gas chromatography–mass spectrometry (GC–MS) was used to determine the fatty acid composition of PSO following a procedure described in a previous study [40]. PSO (100 mg) was weighed into 15 mL glass vials after which 2.0 mL hexane, 50 µL heptadecanoic acid (1000 mg/L, internal standard), and 1.0 mL of 20% (*v*/*v*) H_2_SO_4_ in methanol were successively added. The samples were thoroughly vortexed before incubation at 80 °C for 1 h in an oven. To the cooled the samples, 3 mL of saturated NaCl was added, and the mixture vortexed and centrifuged at 3000 rpm for 3 min. The hexane extracts were transferred into vials for analysis with GC-MS (6890N, Agilent technologies network, Palo Alto, CA, USA) coupled to an Agilent technologies inert XL EI/CI Mass Selective Detector (MSD) (5975B, Agilent Technologies Inc., Palo Alto, CA, USA). Separation of the FAMEs was performed on a polar RT-2560 (100 m, 0.25 mm ID, 0.20 µm film thickness) (Restek, Bellefonte, Pennsylvania, USA) capillary column. Helium was used as the carrier gas at a flow rate of 0.017 mL/s. One microliter (1 µL) of the sample was injected in a split ratio of 10:1. The oven temperature was run as: 100 °C/min, 180 °C at 25 °C/min, and held for 3 min; 200 °C at 4 °C/min and held for 5 min; 280 °C at 8 °C/min, 310 °C at 10 °C/min, and held for 5 min. The PSO fatty acids profiles were identified using the NIST library. Results were expressed as a percentage of the total and calculated by dividing the area peak of each fatty acid by the total area peaks of all the fatty acids.

### 2.9. Statistical Analysis

The results of all the studied variables are presented as mean ± SD (standard deviation). One-way analysis of variance (ANOVA) was performed to compare the means using Statistica software (Statistical v13, TIBC, Palo Alto, CA, USA) after which the means were separated using Duncan’s multiple range test. Graphs were prepared using Microsoft Excel (Version: 16.0.13029.20344, Microsoft Cooperation, Washington, USA). The relationship between the PSO quality attributes and cultivars was determined by performing the principal component analysis (PCA) and agglomerative hierarchical clustering (AHC) and using Microsoft Excel software (XLSTAT 2019.4.1.63305, Addinsoft, New York, NY, USA).

## 3. Results and Discussion

### 3.1. Oil Yield and Seeds Microstructures

**Figure 1:**
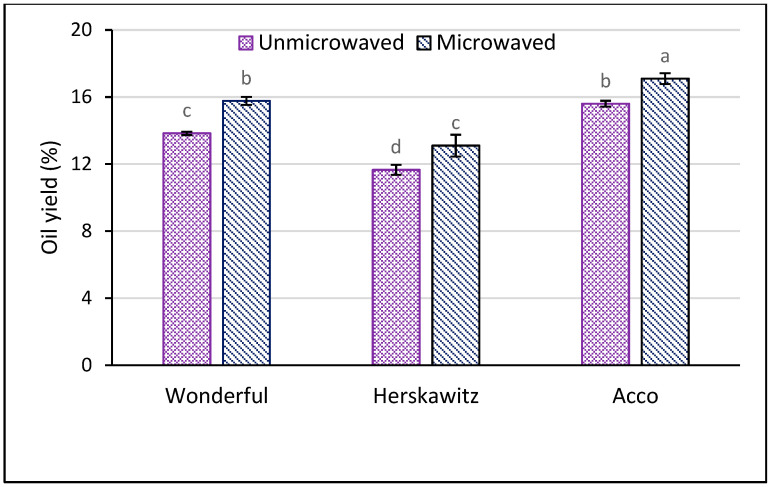
Oil yield from unmicrowaved and microwaved (261 W for 102 s) pomegranate seeds of three pomegranate cultivars. Within the same cultivar (unmicrowaved and microwave), columns followed by different letters are significantly different (*p* < 0.05) according to Duncan’s multiple range test. Vertical bars indicate the standard deviation of the mean.

**Figure 2:**
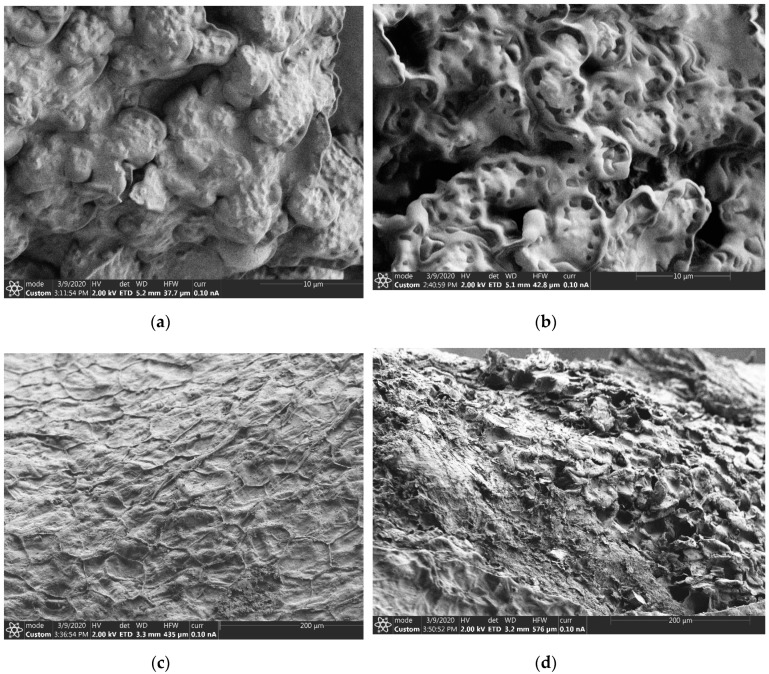
Representative scanning electron microscopy (SEM) micrographs show the effect of microwave pretreatment (261 W/102 s) on the pomegranate seeds microstructures. (**a**) Unmicrowaved pomegranate seeds, (**b**) microwaved pomegranate seeds, (**c**) parenchymal cells from unmicrowaved pomegranate seeds, and (**d**) parenchymal cells from microwaved pomegranate seed.

The results in Figure 1 shows that pretreating pomegranate seeds (PS) with microwaves significantly enhanced the oil yield between 10% and 14%. ‘Acco’ exhibited significantly higher oil yield (17.10%) (dw) than ‘Wonderful’ (15.77%) (dw) and ‘Herskawitz’ (13.10%) (dw) after PS microwave pretreatment, a phenomenon that can be explained by the differences in their genetic material [41]. Previously, Durdevic et al. [19] reported that microwave (100, 250 and 600 W for 2 and 6 min) pretreatment of PS could enhance oil yield between 23% and 32%. Compared to the current study, the difference in oil yield could be explained by variation in cultivar, seeds pretreatment conditions, oil extraction methods, and fruit growing region, among other factors. Depending on the concentration of cellulose and lignin, PS rheological properties such as hardness and toughness may vary with cultivar [42]. This could have affected the cultivars’ response to microwave pretreatment and oil extraction. The scanning electron microscopy (SEM) images in Figure 2 confirmed that PS microwave pretreatment significantly deformed the cell walls. As shown in Figure 2b microwave pretreated PS were characterized by conspicuous perforations on the cell walls. Microwaves generate heat by interacting with polar substances and, therefore, water as a polar molecule is an essential heat transfer medium during seeds microwave pretreatment [30]. The heat energy causes a rapid increase in the seed temperature and vaporization of the water in the seeds creating an intracellular pressure that ruptures the oilseeds cell walls and membranes [20]. Figure 2c shows parenchymal cells from unmicrowaved PS with intact cell walls, which could have created a major resistance to solvent penetration into the seeds cells and could be the reason for the low oil yield observed from unmicrowaved PS [43]. On the other hand, Figure 2d shows extensively damaged PS parenchymal cells due to microwave treatment. Similar findings have been reported from microwave pretreatment of hazelnuts [44]. In addition to damaging the cell walls, microwave pretreatment could have deformed the lipoprotein membranes surrounding the individual lipid bodies [45]. These microstructural changes could have enhanced porosity of the PS cell walls and membranes that led to the improved efficiency of oil extraction with ethanol.

### 3.2. Refractive and Yellowness Index

Thermal treatment of the oilseeds could result in fatty acids conjugation and an increase in the oil refractive index [46]. Therefore, RI could be used as an indirect quality measure of oil. Neither cultivar nor PS microwave pretreatment significantly (*p* > 0.05) affected the RI of the oil extracts, despite the significant cell walls and membranes deformation (Table 1). The pomegranate seed oil RI values (1.5180–1.5181) in the current study were comparable to those reported by Costa et al. [47] (1.5091–1.5177) from cold pressed PSO further demonstrating that PS microwave pretreatment did not cause significant negative effect on the oil quality.

**Figure 3:**
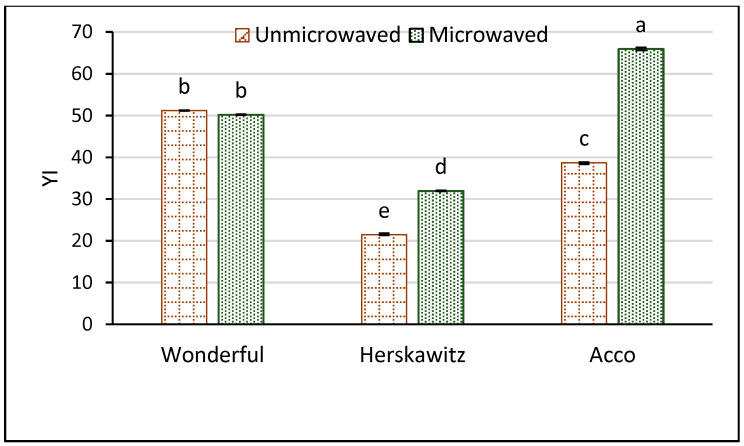
Yellowness index (YI) of pomegranate seed oil from unmicrowaved and microwave pretreated (261 W/102 s) seeds of three pomegranate cultivars. Within the same cultivar (unmicrowaved and microwaved), columns followed by different letters are significantly different (*p* < 0.05) according to Duncan’s multiple range test. Vertical bars indicate the standard deviation of the mean.

Color is a valuable parameter that influences the consumer’s preference and decision to purchase a food product. Yellowness index can be used to measure the influence of processing, including seeds microwave pretreatment on oil color [48]. The results in Figure 3 show that PS microwave pretreatment significantly improved the YI of ‘Herskawitz’ and ‘Acco’ oil extracts by 1.5 and 1.7 fold, respectively. The significant increase in the YI after PS microwave pretreatment could be ascribed to the improved extraction of the oil color pigments such as carotenoids as facilitated by the extensively damaged cell walls and membranes (Figure 2). In the study of Rekas et al. [49], YI significantly increased by 13% and 63% when dehulled rape seeds were microwaved at 800 W for 2 and 4 min, respectively. However, PS microwave pretreatment insignificantly (*p* > 0.05) changed the YI of ‘Wonderful’ oil extracts suggesting that the effect of pretreating PS with microwaves on oil color compounds differed among the cultivars.

### 3.3. Peroxide Value, Conjugated Dienes and Trienes, ρ-Anisidine Value and Total Oxidation Value

Peroxide value indicates the extent of fats and oils oxidation and therefore is one of the most widely used quality indicators in the food industry. As shown in Table 1, PS microwave pretreatment significantly increased the PV from ‘Herskawitz’ and ‘Acco’ oil extracts by 29% and 30%, respectively. The significant increase in PV could be explained by significant heat penetration into the seeds matrices during microwave pretreatment that could have induced lipid oxidation and hydroperoxides formation. Despite the significant increase in PV of ‘Herskawitz’ and ‘Acco’ oil extracts after seeds microwave pretreatment, the values (0.17–0.35 meqO_2_/kg PSO) conformed to the Codex Alimentarius Commission standard for seed oil that permits a maximum of 15 meqO_2_/kg in unrefined seed oils [50]. Microwave pretreatment of PS did not significantly oxidize ‘Wonderful’ oil extracts. Our PV results were lower than those reported by Basiri [51] (0.79 meqO_2_/kg PSO) from petroleum ether extracted PSO, further demonstrating that microwave pretreatment of PS may not cause significant oil degradation.

During hydroperoxides formation, the non-conjugated double bonds of fatty acids may be converted to conjugated double bonds through isomerization [52]. Therefore, fatty acids conjugation can also be used as a quick indirect quality measure of oil. Conjugated dienes are part of fatty acid oxidation initial products. The results in Table 1, demonstrate that the level of K232 significantly increased in ‘Wonderful’ (27%) and ‘Acco’ (45%) oil extracts, whilst it significantly decreased in ‘Herskawitz’ (37%) oil extracts after PS microwave pretreatment. Although there was a significant increase in conjugated dienes in ‘Wonderful’ and ‘Acco’ oil extracts due to PS microwave pretreatment, the K232 values (0.19–0.30) were lower than the K232 values (4.15) reported by Amri et al. [53] from ‘Tounsi’ hexane PSO extracts, indicating that the oil from the present study was of higher quality.

A low PV is not the only marker for good oil quality because hydroperoxides are unstable and quickly decompose into secondary oxidation products. For this reason, the analysis of secondary products of seed oil oxidation is equally important. Unlike the K232 values, PS microwave pretreatment did not significantly influence the levels of K270 values in the oil from all the cultivars. *ρ*-Anisidine value measures the secondary products of fatty acids oxidation, such as aldehydes formed due to further hydroperoxides decomposition [52]. Despite the AV of oil extracted from ‘Acco’ significantly increasing by 3 fold after PS microwave pretreatment, the values (2.00–5.90) were lower than those from ‘Wonderful’ (14.22–12.50) and ‘Herskawitz’ (13.06–12.90) oil extracts that insignificantly changed after PS microwave pretreatment (Table 1). The observation that AV from ‘Wonderful’ and ‘Herskawitz’ oil extracts did not significantly change after PS microwave pretreatment suggests that there was minimum decomposition of hydroperoxides to form carbonyl compounds. In a previous study, Costa et al. [47] reported AV ranging from 13.8 to 18.6 from cold pressed PSO that were higher than the AV (5.90–14.22) results in the present study. High AV in freshly processed seed oil could indicate interference by other substances, leading to false positive overestimation of AV values.

Total oxidation value is a summation of the primary and secondary oxidation products and provides a better indication of fats and oils overall oxidative deterioration. As can be seen in Table 1, PS microwave pretreatment significantly increased the TOTOX value of ‘Acco’ oil extracts by 2.6 fold and this could be linked to either increased heat penetration into the seed matrices or increased lipolytic enzyme activity in the microwave damaged cells [54]. The result that the level of TOTOX value from ‘Wonderful’ and ‘Herskawitz’ oil extracts did not significantly change after PS microwave pretreatment suggests resistance to oxidation by the oil from the two cultivars, which could be attributed to enhanced total phenolic compounds [43].

### 3.4. Total Carotenoids Content, Total Phenolic Content, and Antioxidant Capacity

Epidemiological studies suggest that the consumption of carotenoid-rich foods such as seed oil is associated with the prevention of cancers, cardiovascular diseases, age-related cataracts, and immune system function improvement [55]. Therefore, the maximum extraction of these antioxidative compounds from plant materials such as seeds is important. The results in Table 2 indicate that PS microwave pretreatment significantly increased the total carotenoids content of ‘Herskawitz’ and ‘Acco’ oil extracts by 11% and 19%, respectively. Previously, Mazaheri et al. [56] also observed a significant improvement in carotenoids after microwave pretreatment of black cumin seeds. The extensive damage of the PS cell walls and membranes by microwave pretreatment could have increased the dissociation of carotenoids from the carotenoprotein complexes enhancing their mass transfer into the extraction solvent (Figure 2) [57]. On the other hand, the finding that TCC of oil extracted from ‘Wonderful’ did not significantly change after PS microwave pretreatment, whilst that of ‘Herskawitz’ and ‘Acco’ oil extracts significantly changed after seeds microwave pretreatment, indicating that the response of carotenoids compounds to PS microwave pretreatment was cultivar dependent.

Phenolic compounds have been implicated in the anti-inflammatory and antioxidant properties of potential functional foods [26]. In this respect, polyphenol-rich foods intake may be associated with decreased risk of chronic diseases. PS microwave pretreatment significantly enhanced the total phenolic compounds of oil extracted from ‘Wonderful’ and ‘Acco’ by 25% and 17%, respectively, but did not significantly change the TPC of ‘Herskawitz’ oil extracts (Table 2). The results demonstrate that cultivar is an invaluable factor in PSO value addition. In addition, it has been reported that in plant materials phenolic compounds exist as either glycosylated, non-glycosylated, esterified, or free compounds, which could vary with cultivar and significantly influence their extraction [58]. The TPC (1.67–3.12 mg GAE/g PSO) results from the current study were higher than those reported by Pande and Akoh [59] (0.85–0.91 mg/g PSO) and Costa et al. [47] (0.00–0.17 mg/g PSO) from solvent extracted, and cold pressed PSO an indication that pomegranate cultivars from the current study could be valuable sources of phenolic compounds. Besides, factors such as cultivar, oil extraction technique, and fruit growing region could also be sources of variation in the TPC results among the studies.

Phytochemicals are complex; no single assay accurately reflects all antioxidants in a complex system such as seed oil. In this study, the antioxidant capacity of PSO was assessed using the DPPH and FRAP assays. PS microwave pretreatment significantly enhanced the DPPH radical scavenging capacity of ‘Herskawitz’ (7%) and ‘Acco’ (4%) oil extracts but did not significantly change the DPPH radical scavenging capacity of oil extracted from ‘Wonderful’. FRAP significantly increased in the oil extracted from ‘Wonderful’ and ‘Herskawitz’ by 47 and 82%, respectively, after PS microwave pretreatment. In contrast to this finding, the reducing potential of ‘Acco’ oil extracts did not significantly change after PS microwave pretreatment. The finding that cultivar significantly influenced the antioxidant capacity of oil from microwave pretreated PS agrees with the results from Xi et al. [60], who reported significant variation in DPPH radical scavenging capacity and FRAP of seed oil from different lemon cultivars. The significant increase in the oil antioxidant capacity after PS microwave pretreatment, particularly from ‘Herskawitz’ could be related to the improved TCC and TPC. However, it is worth mentioning that PSO is also a good source of tocopherols, which have been reported to be associated with the oil antioxidant capacity in previous studies [61].

### 3.5. Phytosterol Composition

**Figure 4:**
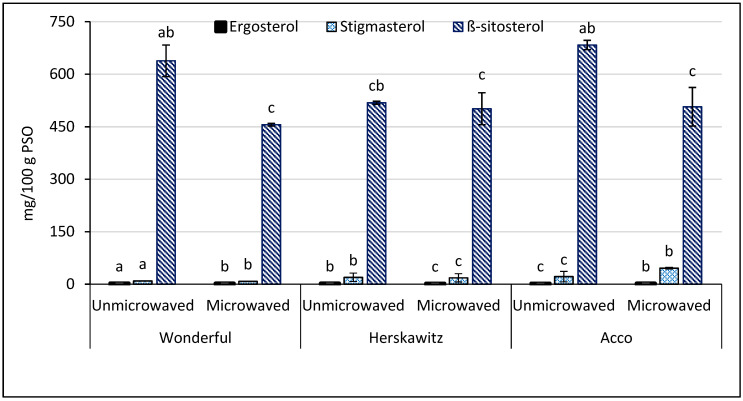
Phytosterol composition of oil extracted from unmicrowaved and microwave pretreated (261 W/102 s) seeds of three pomegranate cultivars. Within the same cultivar (unmicrowaved and microwaved), columns representing the same phytosterol and followed by different letters are significantly different (*p* < 0.05) according to Duncan’s multiple range test. Vertical bars indicate the standard deviation of the mean.

The ability of phytosterols to lower blood cholesterol may reduce the risk of coronary heart disease. Optimum extraction of these valuable compounds during seed oil processing is therefore essential to enhance the extracted oil health benefits. The effect of PS microwave pretreatment on phytosterol composition is presented in Figure 4. Three different phytosterols, including β-sitosterol (455.91–683.37 mg/100g PSO), stigmasterol (9.04–45.74 mg/100g PSO), and ergosterol (2.06–2.53 mg/100g PSO) were quantified in PSO from the studied cultivars. The levels of β-sitosterol and stigmasterol were consistent with the findings of Pande and Akoh [59] and Caligiani et al. [62] from PSO extracted using hexane and ethyl ether, respectively. In addition, the concentration of phytosterols was higher than those reported from other fruit seed oils such as apple, strawberry, and raspberry but comparable to those from sour cherry [63,64]. The level of β-sitosterol significantly decreased in ‘Acco’ and ‘Wonderful’ oil extracts by 26% and 29%, respectively, after PS microwave pretreatment, whilst it did not significantly change in ‘Herskawitz’ oil extracts, regardless of the PS cell walls and membranes extensive damage by microwave pretreatment (Figure 2). The finding that β-sitosterol significantly decreased in ‘Wonderful’ and ‘Acco’ oil extracts after PS microwave pretreatment suggests that the applied microwave pretreatment conditions thermally degraded this low-density lipoprotein (LDL) reducing phytosterol. Likewise, the levels of stigmasterol and ergosterol significantly decreased between 8 and 13% in ‘Wonderful’ and ‘Herskawitz’ oil extracts after treating the seeds with microwaves. Unlike in the present study, Azadmard-Damirchi et al. [21] and Fathi-Achachlouei et al. [43] reported significant improvement in phytosterols from microwave pretreated rape and milk thistle seed, respectively. The dissimilarity of our results with previous studies indicates that microwave pretreatment conditions are seed specific.

On the other hand, microwave pretreatment of PS significantly improved the concentration of ergosterol and stigmasterol (9 and 111%, respectively) in ‘Acco’ oil extracts. Naturally, phytosterols exist as free compounds or conjugates in which they are either esterified to fatty acids or glycosylated with sugars [65]. The form in which they exist may therefore influence their dissociation and isolation from the seed matrix.

### 3.6. Fatty Acid Composition

**Figure 5:**
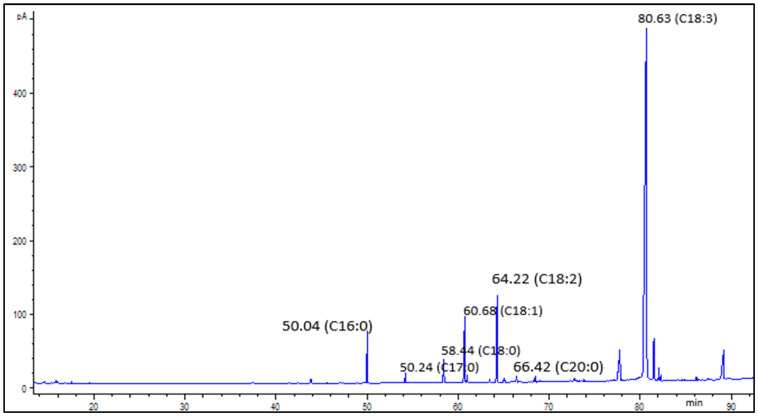
***A*** typical gas chromatography-mass spectrometry (GC-MS) chromatograph of the major fatty acids identified in pomegranate seed oil and their retention times. C16:0 = palmitic acid, C17:0 = heptadecanoic acid (internal standard), C18:0 = stearic acid, C18:1 = oleic acid, C18:2 = linoleic acid, C18:3 = punicic acid, C20:0 = arachidic acid.

The GC chromatogram shows that the primary fatty acids identified in PSO from the studied cultivars were palmitic acid, stearic acid, oleic acid, linoleic acid and punicic acid (Figure 5), which accounted for 5.64–7.74%, 2.34–3.08%, 7.43–9.62%, 11.59–16.54%, and 62.75–70.51%, respectively. The fatty acid composition was comparable to the findings of Tian et al. [66] and Aruna et al. [67]. However, the values of punicic acid were lower when compared with the findings of Khoddami et al. [5] and Fernandes et al. [4], which could be attributed to differences in fruit ripening index, seed oil extraction method, cultivar, and geographical location, among other factors. As can be seen in Table 3, PS microwave pretreatment significantly increased palmitic acid between 6% and 20%. Moreover, treatment of PS with microwaves significantly increased the stearic acid from ‘Acco’ oil extracts by 7%. Among the saturated fatty acids, stearic acid has unique properties, as it has been associated with a decrease in LDL cholesterol, cancer, and atherosclerosis risk [68]. The levels of stearic acid did not significantly change in ‘Wonderful’ and ‘Herskawitz’ oil extracts after PS microwave irradiation, which was comparable with the results reported by Durdevic et al. [69]. Oleic acid, the main monosaturated fatty acid in PSO significantly improved in ‘Herskawitz’ and ‘Acco’ oil extracts by 9% and 10%, respectively, after seeds microwave pretreatment. Improvement in oleic acid after seeds microwave pretreatment is desirable in oil oxidative stability as monosaturated fatty acids are less susceptible to oxidation. More so, PS microwave pretreatment significantly increased linoleic acid from ‘Herskawitz’ (37%) and ‘Acco’ (12%) oil extracts. The finding that PS microwave pretreatment enhanced the oil oleic acid and linoleic acid is essential to human health since oleic acid is associated with lowering low density lipoprotein (LDL) blood cholesterol, and linoleic acid has an important role in balancing fatty acid content proportions in body cells [20]. However, the concentration of punicic acid, the primary bioactive lipid with several biological properties significantly decreased in ‘Herskawitz’ and ‘Acco’ by 10% and 5%, respectively (Table 3). PSO health benefits potential is mostly attributed to punicic acid and therefore, its decrease after PS microwave pretreatment was not desirable. Arachidic acid significantly increased in ‘Herskawitz’ (13%) oil extracts, whilst it significantly decreased in ‘Acco’ (14%) oil extracts. Saturated fatty acids (SFA) significantly increased in ‘Acco’ (11%) and ‘Herskawitz’ (15%) oil extracts after PS microwave pretreatment. However, the polyunsaturated fatty acids (PUFA) and the ratio of unsaturated to saturated fatty acid (UFA: SFA) in ‘Herskawitz’ oil extracts decreased by 3% and 4%, respectively, after PS microwave pretreatment, indicating a loss in nutritional quality despite the significant increase in the antioxidant capacity (Table 1 and Table 2). The decrease in the ratio of UFA: SFA could be attributed to the decline in punicic acid, the main polyunsaturated fatty acid in PSO. The amount of monosaturated fatty acids (MUFA) was insignificantly changed after microwave pretreatment of PS from all the cultivars. The levels of PUFA and UFA: SFA ratio in oil extracted from ‘Acco’ were not significantly affected by PS microwave pretreatment. Except for palmitic acid, PS microwave pretreatment had no significant effect (*p* > 0.05) on the fatty acid content of oil extracted from ‘Wonderful’. Insignificant effect of seeds microwave pretreatment on the oil fatty acids content has also been reported in prior researches. For example, Wroniak et al. [20] and Guneser and Yilmaz [25] observed no significant change in the fatty acids content of oil extracted from orange and rape seeds, respectively, after microwave pretreatment.

### 3.7. Principal Component Analysis and Agglomerative Hierarchical Clustering Analysis

**Figure 6:**
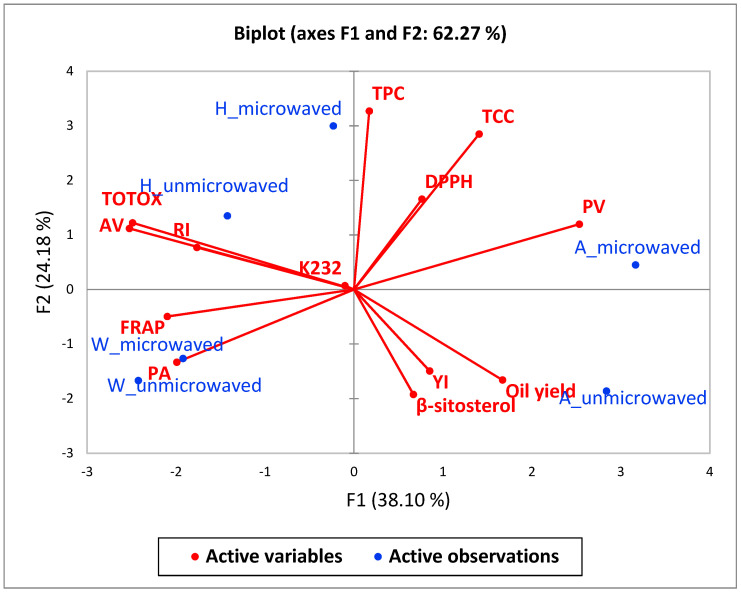
Principal component analysis data of pomegranate seed oil (PSO) quality attributes from unmicrowaved and microwaved (261 W/102 s) pomegranate seeds of three pomegranate cultivars. A = ‘Acco’, H = ‘Herskawitz’, W = ‘Wonderful’, AV = *ρ*-anisidine value, TOTOX = Total oxidation value, K232 = Conjugated dienes, RI = Refractive index, TPC = Total phenolic content, TCC = Total carotenoids content, PA = punicic acid, FRAP = Ferric reducing antioxidant power, DPPH = 2,2-diphenyl-1-picryl hydrazyl.

**Figure 7:**
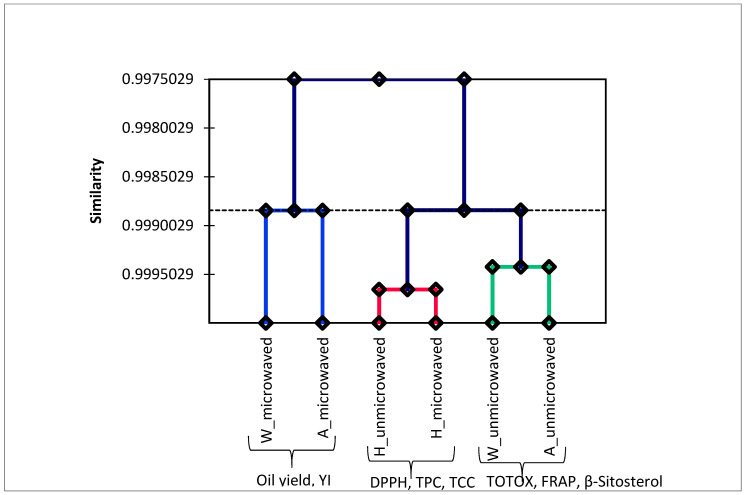
Agglomerative hierarchical clustering (AHC) of PSO extracts from unmicrowaved and microwaved (261 W/102 s) seeds. A = ‘Acco’, H = ‘Herskawitz’, W = ‘Wonderful’, TOTOX = Total oxidation value, TPC = Total phenolic content, TCC = Total carotenoids content, DPPH = 2,2-diphenyl-1-picryl hydrazyl, FRAP = Ferric reducing antioxidant power.

Principal component analysis (PCA) and agglomerative hierarchical clustering (AHC) were performed in order to provide an overview of the relationship between pomegranate cultivars, seeds microwave pretreatment, and the oil quality attributes. According to Kaiser’s rule, only eigenvalues greater than 1 are considered significant descriptors of data variance [70]. The first two factors with the highest eigenvalues (F1 = 5.0, F2 = 3.1) accounted for 62.27% (F1: 38.10% and F2: 24.10, respectively) of the total variance in the original data and were considered more important (Figure 6). The first factor (F1), which was contributed by ‘Acco’ and ‘Wonderful’ oil extracts, was positively correlated with oil yield and PV, but negatively correlated with RI, AV, TOTOX, punicic acid, and FRAP. This points out that cultivars, which exhibited higher oil yield after seeds microwave pretreatment such as ‘Acco’, were associated with low FRAP. Although extensive damage of the PS cell walls and membranes by microwave pretreatment facilitated increased extraction of lipids, it could have exposed the oil to thermal degradation (Figure 2). The second factor (F2) that was contributed by ‘Herskawitz’ oil extracts from microwave pretreated seeds and ‘Acco’ oil extracts from unmicrowaved seeds was positively correlated with TCC, TPC, and DPPH radical scavenging capacity, but negatively correlated with β-sitosterol and YI. As shown in Table 2. microwave pretreatment of ‘Herskawitz’ PS improved the extraction of TCC and TPC that could have enhanced the oil DPPH radical scavenging capacity. The agglomerative hierarchical clustering (AHC) of PSO extracts from the different cultivars clustered the ‘Acco’ and ‘Wonderful’ oil extracts from microwaved seeds together that were correlated with higher oil yield and YI (Figure 7). These results concurred with the PCA analysis results. Like the PCA results, AHC separated ‘Herskawitz’ oil extracts from ‘Acco’ and ‘Wonderful’, illustrating that ‘Herskawitz’ was higher in oil quality attributes that were lower in ‘Acco’ and ‘Wonderful’, such as TCC, TPC, and DPPH radical scavenging capacity. Although microwave pretreatment of ‘Acco’ and ‘Wonderful’ PS may enhance oil yield, It was established that it also increases the oil oxidative degradation. On the other hand, microwave pretreatment of ‘Herskawitz’ PS may not produce oil yield results comparable to ‘Wonderful’ and ‘Acco’, but the oil has better bioactive compounds and antioxidant capacity.

## 4. Conclusions

The present study established that PSO quality may be enhanced by seeds during microwave pretreatment, although oil quality varies with cultivar. Microwave pretreatment of PS improved oil yield, YI, TCC, TPC, DPPH radical scavenging capacity, and FRAP. This is a desirable development to the food industry given the increasing consumers’ demand for natural and healthier foods. Moreover, the enhancement of bioactive compounds and antioxidant capacity after PS microwave pretreatment is valuable for the oil oxidative stability and storability. Despite an increase in K232 and PV, PS microwave pretreatment slightly decreased the oil TOTOX value.

Regarding fatty acid composition, PS microwave pretreatment increased palmitic acid, oleic acid, linoleic acid, SFA, and MUFA, but reduced the level of punicic acid. Pretreating PS with microwaves did not significantly affect the RI, PUFA, ratio of UFA: SFA, and phytosterol composition of the extracted oils. According to the PCA and AHC, ‘Acco’ and ‘Wonderful’ oil extracts from microwave pretreated PS exhibited better oil yield, whilst ‘Herskawitz’ oil extracts showed higher TCC, TPC, and DPPH radical scavenging valuable in functional foods formulation. In conclusion, ‘Herskawitz’ is a desirable cultivar for exploitation in nutraceutical and functional foods formulations.

## Figures and Tables

**Table 1:** Physicochemical characteristics of oil from unmicrowaved and microwave pretreated (261 W/102 s) pomegranate seeds of three pomegranate cultivars.

Cultivar	Treatment	RI	K232	K270	PV	AV	TOTOX
Wonderful	Unmicrowaved	1.5181 ± 0.00 ^a^	0.22 ± 0.01 ^b^	0.28 ± 0.005 ^b^	0.04 ± 0.001 ^d^	14.22 ± 0.58 ^a^	14.30 ± 0.58 ^a^
	Microwaved	1.5180 ± 0.00 ^a^	0.28 ± 0.01 ^a^	0.29 ± 0.009 ^ab^	0.05 ± 0.005 ^d^	12.50 ± 0.59 ^a^	12.59 ± 0.59 ^a^
Herskawitz	Unmicrowaved	1.5180 ± 0.00 ^a^	0.30 ± 0.01 ^a^	0.31 ± 0.008 ^a^	0.17 ± 0.008 ^e^	13.06 ± 0.34 ^a^	13.40 ± 0.35 ^a^
	Microwaved	1.5180 ± 0.00 ^a^	0.19 ± 0.02 ^b^	0.29 ± 0.016 ^ab^	0.22 ± 0.011 ^c^	12.90 ± 1.20 ^a^	13.33 ± 1.19 ^a^
Acco	Unmicrowaved	1.5180 ± 0.00 ^a^	0.20 ± 0.01 ^b^	0.31 ± 0.005 ^a^	0.27 ± 0.005 ^a^	2.00 ± 0.66 ^c^	2.53 ± 0.65 ^c^
	Microwaved	1.5180 ± 0.00 ^a^	0.29 ± 0.01 ^a^	0.31 ± 0.009 ^ab^	0.35 ± 0.007 ^b^	5.90 ± 1.15 ^b^	6.60 ± 1.16 ^b^

Values represent mean ± SD of triplicate determinations. Different superscript letters in the same column indicate statistical significance (*p* < 0.05) according to Duncan’s multiple range test. RI= index (25 °C), PV = Peroxide value (meqO_2_/kg PSO), meqO_2_/kg = milli-equivalents of active oxygen per kg), AV = Anisidine value, TOTOX = Total oxidation value, RI = Refractive, K232 = Conjugated dienes, K270 = Conjugated triene.

**Table 2:** TCC, TPC and antioxidant capacity (DPPH, FRAP) of oil extracted from unmicrowaved and microwave pretreated (261 W/102 s) seeds of three pomegranate cultivars.

Cultivar	Treatment	TCC	TPC	DPPH	FRAP
Wonderful	Unmicrowaved	22.65 ± 0.96 ^d^	1.67 ± 0.01 ^c^	1.70 ± 0.05 ^bc^	6.09 ± 1.44 ^b^
	Microwaved	21.19 ± 1.81 ^d^	2.09 ± 0.17 ^b^	1.72 ± 0.02 ^bc^	8.98 ± 0.41 ^a^
Herskawitz	Unmicrowaved	30.27 ± 0.36 ^b^	2.91 ± 0.11 ^a^	1.66 ± 0.01 ^c^	3.00 ± 0.17 ^c^
	Microwaved	33.47 ± 0.43 ^a^	3.12 ± 0.07 ^a^	1.78 ± 0.01 ^ab^	5.46 ± 0.90 ^b^
Acco	Unmicrowaved	27.00 ± 0.96 ^c^	2.05 ± 0.06 ^c^	1.69 ± 0.03 ^c^	1.95 ± 0.02 ^c^
	Microwaved	32.08 ± 0.73 ^ab^	2.39 ± 0.13 ^b^	1.76 ± 0.02 ^a^	1.80 ± 0.13 ^c^

Values represent mean ± SD of triplicate determinations. Different superscript letters in the same column indicate statistical significance (*p* < 0.05) according to Duncan’s multiple range test. TPC = Total phenolic content (mg GAE/g PSO, TCC = Total carotenoids content (mg β-carotene/100 g PSO), FRAP = Ferric reducing antioxidant power (mmol Trolox/g PSO), DPPH = 2,2-Diphenyl-1-picryl hydrazyl (mmol Trolox/g PSO), Trolox = 6-hydroxy-2,5,7,8-tetramethylchroman-2-carboxylic acid, PSO = Pomegranate seed oil, GAE = Gallic acid equivalence.

**Table 3:** Fatty acid composition (% relative area) of pomegranate seed oil from unmicrowaved and microwave pretreated (261 W/102 s) seeds of three pomegranate cultivars.

Cultivar/Treatment						
Fatty Acid	Wonderful		Herskawitz		Acco	
	Unmicrowaved	Microwaved	Unmicrowaved	Microwaved	Unmicrowaved	Microwaved
Palmitic acid (C16:0)	5.64 ± 0.14 ^c^	5.98 ± 0.17 ^bc^	5.66 ± 0.35 ^c^	6.82 ± 0.53 ^ab^	6.72 ± 0.16 ^b^	7.74 ± 0.27 ^a^
Stearic acid (C18:0)	2.50 ± 0.08 ^c^	2.49 ± 0.09 ^bc^	2.34 ± 0.11 ^ab^	2.35 ± 0.08 ^ab^	2.87 ± 0.03 ^b^	3.08 ± 0.02 ^a^
Oleic acid (C18:1)	8.04 ± 0.47 ^c^	8.59 ± 0.16 ^bc^	7.43 ± 0.30 ^c^	8.11 ± 0.40 ^ab^	8.75 ± 0.12 ^b^	9.62 ± 0.17 ^a^
Linoleic acid (C18:2)	11.59 ± 0.23 ^c^	11.62 ± 0.53 ^bc^	12.09 ± 1.25 ^c^	16.54 ± 1.53 ^ab^	12.86 ± 0.42 ^b^	14.35 ± 0.95 ^a^
Punicic acid (C18:3)	68.95 ± 0.63 ^c^	68.99 ± 0.71 ^bc^	70.51 ± 1.96 ^c^	63.55 ± 2.84 ^ab^	66.30 ± 0.58 ^b^	62.75 ± 1.84 ^a^
Arachidic acid (C20:0)	0.45 ± 0.03 ^c^	0.54 ± 0.01 ^bc^	0.53 ± 0.02 ^c^	0.60 ± 0.04 ^ab^	0.88 ± 0.19 ^b^	0.76 ± 0.02 ^a^
SFA	8.59 ± 0.24 ^cd^	9.01 ± 0.27 ^cd^	8.53 ± 0.30 ^d^	9.77 ± 0.63 ^bc^	10.47 ± 0.36 ^ab^	11.58 ± 0.30 ^a^
MUFA	8.04 ± 0.47 ^bc^	8.59 ± 0.16 ^b^	7.43 ± 0.30 ^c^	8.11 ± 0.40 ^bc^	8.75 ± 0.12 ^ab^	9.62 ± 0.17 ^a^
PUFA	80.53 ± 0.43 ^ab^	80.61 ± 0.19 ^ab^	82.60 ± 0.70 ^a^	80.09 ± 1.31 ^b^	79.16 ± 0.17 ^bc^	77.09 ± 0.90 ^c^
UFA/SFA ratio	17.43 ± 0.25 ^a^	17.55 ± 0.14 ^a^	17.15 ± 0.15 ^a^	16.40 ± 0.27 ^b^	16.33 ± 0.14 ^b^	16.29 ± 0.08 ^b^

Values represent mean ± SD of triplicate determinations. Different superscript letters in the same row indicate statistical significance (*p* < 0.05) according to Duncan’s multiple range test. SFA = Saturated fatty acid, MUFA = Monounsaturated fatty acid, PUFA = Polyunsaturated fatty acid, UFA = Unsaturated fatty acid.
